# Phosphatase activity of the control of virulence sensor kinase CovS is critical for the pathogenesis of group A *streptococcus*

**DOI:** 10.1371/journal.ppat.1007354

**Published:** 2018-10-31

**Authors:** Nicola Horstmann, Chau Nguyen Tran, Chelcy Brumlow, Sruti DebRoy, Hui Yao, Graciela Nogueras Gonzalez, Nishanth Makthal, Muthiah Kumaraswami, Samuel A. Shelburne

**Affiliations:** 1 Department of Infectious Diseases, Infection Control and Employee Health, MD Anderson Cancer Center, Houston TX, United States of America; 2 Department of Bioinformatics and Computational Biology, MD Anderson Cancer Center, Houston TX, United States of America; 3 Department of Pathology and Genomic Medicine, Houston Methodist Hospital, Houston, TX, United States of America; 4 Department of Genomic Medicine, MD Anderson Cancer Center, Houston TX, United States of America; Boston Children's Hospital, UNITED STATES

## Abstract

The control of virulence regulator/sensor kinase (CovRS) two-component system is critical to the infectivity of group A *streptococcus* (GAS), and CovRS inactivating mutations are frequently observed in GAS strains causing severe human infections. CovS modulates the phosphorylation status and with it the regulatory effect of its cognate regulator CovR via its kinase and phosphatase activity. However, the contribution of each aspect of CovS function to GAS pathogenesis is unknown. We created isoallelic GAS strains that differ only by defined mutations which either abrogate CovR phosphorylation, CovS kinase or CovS phosphatase activity in order to test the contribution of CovR phosphorylation levels to GAS virulence, emergence of hypervirulent CovS-inactivated strains during infection, and GAS global gene expression. These sets of strains were created in both serotype M1 and M3 backgrounds, two prevalent GAS disease-causing serotypes, to ascertain whether our observations were serotype-specific. In both serotypes, GAS strains lacking CovS phosphatase activity (CovS-T284A) were profoundly impaired in their ability to cause skin infection or colonize the oropharynx in mice and to survive neutrophil killing in human blood. Further, response to the human cathelicidin LL-37 was abrogated. Hypervirulent GAS isolates harboring inactivating CovRS mutations were not recovered from mice infected with M1 strain M1-CovS-T284A and only sparsely recovered from mice infected with M3 strain M3-CovS-T284A late in the infection course. Consistent with our virulence data, transcriptome analyses revealed increased repression of a broad array of virulence genes in the CovS phosphatase deficient strains, including the genes encoding the key anti-phagocytic M protein and its positive regulator Mga, which are not typically part of the CovRS transcriptome. Taken together, these data establish a key role for CovS phosphatase activity in GAS pathogenesis and suggest that CovS phosphatase activity could be a promising therapeutic target in GAS without promoting emergence of hypervirulent CovS-inactivated strains.

## Author summary

Group A *streptococcus* (GAS), also known as *Streptococcus pyogenes*, causes a broad array of human infections of varying severity. Tight control of production of virulence factors is critical to GAS pathogenesis, and the control of virulence two-component signaling system (CovRS) is central to this process. The activity of the bifunctional histidine kinase CovS determines the phosphorylation status and thereby the activity of its cognate response regulator CovR. Herein, we sought to determine how varying CovR phosphorylation level (CovR~P) impacts GAS pathophysiology. Using three infection models, we discovered that GAS strains lacking CovS phosphatase activity resulting in high CovR~P levels had markedly impaired infectivity. Transcriptome analysis revealed that the hypovirulent phenotype of CovS phosphatase deficient strains is due to down-regulation of numerous genes encoding GAS virulence factors. We identified repression of additional virulence genes that are typically not controlled by CovR, thus expanding the CovR regulon at high CovR~P concentrations. Our data indicate that phosphatase activity of CovS sensor kinase is crucial for spatiotemporal regulation of GAS virulence gene expression. Thus, we propose that targeting the phosphatase activity of CovS sensor kinase could be a promising novel therapeutic approach to combat GAS disease.

## Introduction

The ability of bacteria to modify gene expression levels in adaptation to external influences is key to many aspects of bacterial pathogenesis [1]. Two-component regulatory systems (TCS) are a major mechanism by which bacteria detect and respond to diverse environmental factors [2, 3]. TCS are absent in humans but abundant in a wide range of bacteria. They usually consist of a membrane-embedded histidine kinase that determines the regulatory activity of its cognate response regulator by altering its phosphorylation status [2, 4].

The control of virulence regulator sensor (CovRS, also called CsrRS for capsule synthesis regulator) system of group A *streptococcus* (GAS) is one of the best-studied TCS in connection with bacterial pathogenesis [5, 6]. GAS is a strictly human pathogen that causes a variety of diseases from relatively benign to life threatening such as necrotizing fasciitis [7]. GAS strains are classified into >200 serotypes based on variability in the key anti-phagocytic, cell-surface exposed M protein [8, 9]. In tandem with the histidine kinase CovS, CovR is the central regulator of GAS virulence factor production [5, 10]. Similar to other OmpR/PhoB family members, CovR is phosphorylated at a conserved aspartic acid residue (D53) to create CovR~P, which is considered to be the active regulatory form of the protein [11–13].

Several signaling pathways converge to tightly regulate CovR~P levels. CovS primarily serves to increase CovR~P via its kinase activity [14]. As a member of the bifunctional HisKA-family of histidine kinases [15], CovS also possesses phosphatase activity to reduce CovR~P. Extracellular signals (e.g. Mg^2+^ or LL-37) influence CovS activity to modulate CovR~P levels and CovR-regulated virulence gene expression [16–18]. Deletion of *covS* reduces but does not completely eliminate CovR~P [14] suggesting a contribution of intracellular metabolites such as acetyl phosphate to CovR phosphorylation. In combination with CovS, the orphan kinase regulator of CovR, RocA, increases CovR~P levels [19]. It has been discovered that conserved mutations in the *rocA* gene mediate serotype-specific intrinsic CovR~P levels [20, 21]. Finally, serine/threonine kinase (Stk) phosphorylates CovR residue threonine 65 in a fashion that antagonizes D53 phosphorylation [22]. This multi-faceted regulation of CovR~P status underpins its key position in GAS infectivity, which is further highlighted by the observation that the CovRS system is one of the hotspots for mutations in invasive clinical isolates [23, 24]. Given that CovR mainly serves as a transcriptional repressor, emergence of CovRS inactivating mutations during human infection or animal passage result in hypervirulent GAS strains due to relieved repression of virulence gene expression [25–28]. It has been speculated that there is a serotype/strain-specific tendency for the acquisition of *covRS* mutations that comes along with the underlying invasive potential of different M-types [29]. This tendency is thought to be determined by serotype-specific expression of certain virulence factors, such as the hyaluronic acid capsule, the M protein and the secreted DNase Sda1 [30–33]. Likewise, the intrinsic CovR~P levels of GAS strains might play a role in determining the selective pressure to lower CovR~P via *covRS* mutations [14].

Although the CovRS system has been extensively studied, several key outstanding issues regarding its impact on GAS pathogenesis remain unanswered. First, it is well established that CovRS-inactivation increases GAS virulence in bacteremia models [5, 21, 22, 25, 34]. However, there are conflicting results regarding how CovR~P levels impact GAS skin/soft tissue infection and nasopharyngeal colonization [20, 25, 26, 35, 36]. Second, the specific influence of CovR kinase and phosphatase activity on GAS virulence is not clear since previous research has focused on the impact of CovR or CovS inactivation. We recently showed that a strain lacking CovS phosphatase activity resulted in increased CovR~P levels [14]. However, how increased CovR~P levels due to abrogating CovS phosphatase activity impact GAS infectivity remains unknown. Finally, virulence studies using strains with varying CovR~P levels have generally been restricted to a single GAS serotype leaving open the question of the applicability of findings in a single strain to the broader GAS population. To address these questions, we employed isoallelic GAS strains of two distinct M serotypes that are common causes of human disease to comprehensively investigate how defined CovR~P levels impact GAS virulence, global gene expression, and emergence of hypervirulent CovRS mutated strains.

## Results

### Generation of serotype M1 strains with defined CovR phosphorylation levels

**Fig 1:**
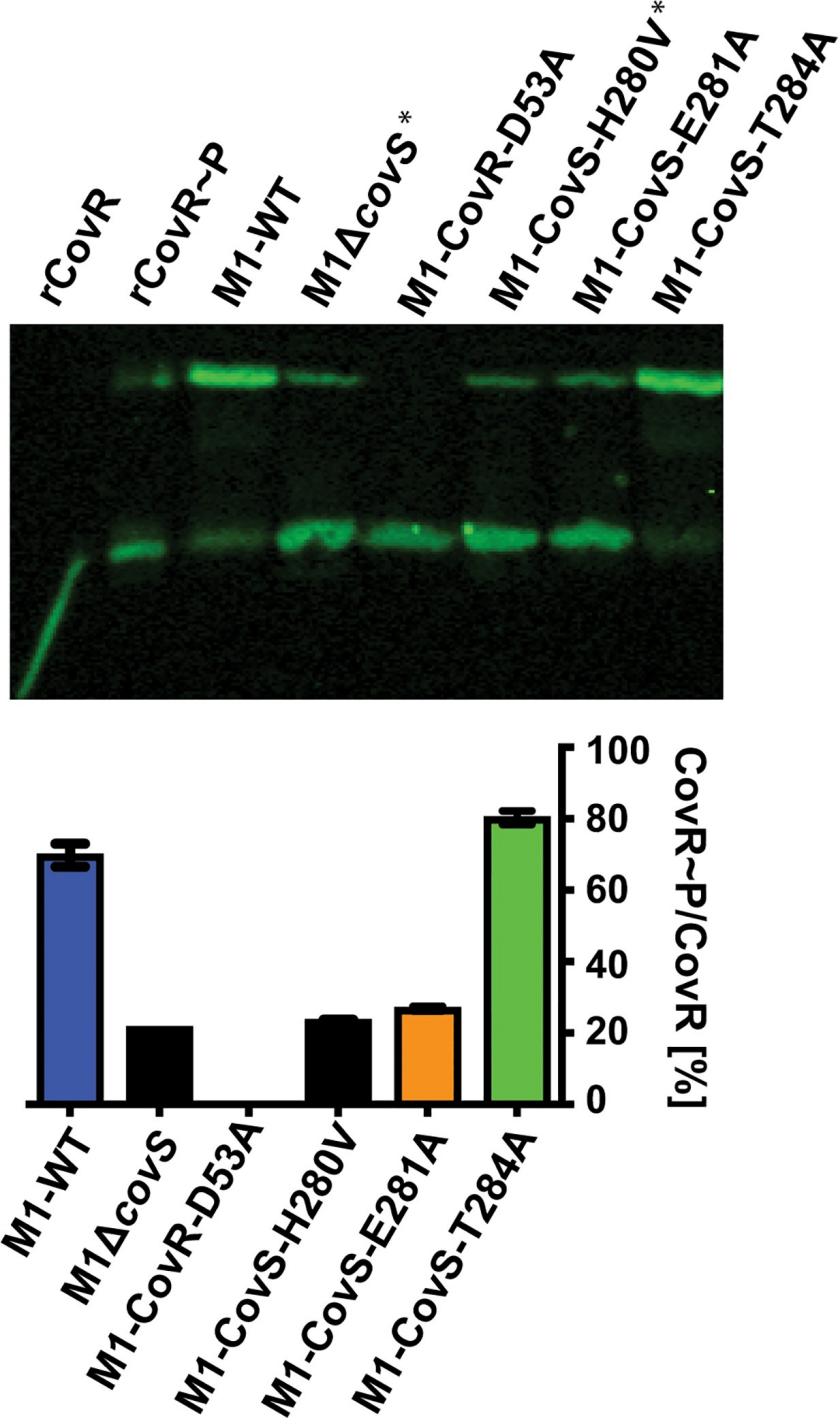
CovR~P levels in serotype M1 GAS strains. Representative Western blot (n = 2) of 10 μg total protein (top) and bar representation (bottom) depicting CovR~P levels (CovR~P/CovR_total_) in cell lysates of indicated strains. Unphosphorylated and phosphorylated (retarded band) CovR species were separated by 12.5% Zn^2+^-Phostag SDS-PAGE. Recombinant CovR (rCovR) in absence or presence of acetyl-phosphate served as controls. Error bars represent standard deviation of the means. Strains M1-Δ*covS* (no functional CovS) and M1-CovS-H280V (CovS autophosphorylation site) (*, black bars) were not further used in this study.

We have previously created and characterized isoallelic serotype M3 strains in which single amino acids in CovRS are altered compared to the parental strain MGAS10870 [14, 22]. We recreated the same set of mutations in the parental strain MGAS2221, a contemporary serotype M1 GAS strain (see Table 1) (For clarity of the genetic background of the used strains, we refer to these strains as M1-wild type (WT), M3-wild type (WT) etc. for the remainder of the manuscript). The CovR~P levels (defined as [CovR~P]/[CovR]total) in the respective M1 GAS strains during growth in THY medium were determined by Phostag-Western blot using anti-CovR antibodies (Fig 1). Similar to our M3 studies [14, 22], changing the CovR phosphorylation site aspartate 53 to an alanine resulted in no detectable CovR~P in strain M1-CovR-D53A. Abolishing CovS kinase activity via the E281A mutation decreased CovR~P levels to ~20% in M1-CovS-E281A, resembling a *covS* deletion strain, whereas the T284A change abrogating the CovS phosphatase activity increased CovR~P levels to ~80% in strain M1-CovS-T284A (Fig 1). Thus, the CovR~P levels observed in the isoallelic mutant strains of M1-WT closely mirrored those previously observed for the serotype M3 derivatives [14]. However, it is important to note that CovR~P levels are higher in the M1-WT strain (~70%) relative to the M3-WT strain (~40%) due to a naturally occurring mutation in the RocA protein in M3 strains [14, 21]. This collection of strains allowed us to study in detail the impact of CovR phosphorylation on GAS virulence and *covRS* switching rates during infection in two of the most prevalent GAS serotypes causing pharyngitis and severe infections in the US (Center for Disease Control and Prevention [37]).

**Table 1:** Strains used in this study.

Strains with distinct CovR~P levels (%)#	Description	Reference
M3-WT (~40)	Clinical isolate (M3); *covRS* wild type, RocA negative	[23]
M3-Δ*covR* (0)	MGAS10870, Δ*covR*:*aphA3*	[28]
M3-CovR-D53A (0)	MGAS10870 with *covR* encoding Ala at position 53	[22]
M3-Δ*covS* (~20)	MGAS10870, Δ*covS*:*spec*	[22]
M3-CovS-E281A (~20)	MGAS10870 with *covS* encoding Ala at position 281	[14]
M3-CovS-T284A (~70)	MGAS10870 with *covS* encoding Ala at position 284	[14]
M1-WT (~70)	Clinical isolate (M1); *covRS* wild type, RocA wild-type	[25]
M1-CovR-D53A (0)	MGAS2221 with *covR* encoding Ala at position 53	This study
M1- Δ*covS* (~20)	MGAS2221 with 7 bp insertion in *covS*	[38]
M1-CovS-H280V (~20)	MGAS2221 with *covS* encoding Val at position 280	This study
M1-CovS-E281A (~20)	MGAS2221 with *covS* encoding Ala at position 281	This study
M1-CovS-T284A (~80)	MGAS2221 with *covS* encoding Ala at position 284	This study
M1-*rocA*^M3^ (~40)	MGAS2221 with *rocA* sequence of M3 (truncated RocA)	This study
M1-*rocA*^M3^-CovS-T284A (~80)	MGAS2221 with *rocA* sequence of M3 (truncated RocA) and *covS* encoding Ala at position 284	This study
**Isoallelic strains harboring SNPs found in virulence study** CovR-R66H	MGAS10870 with *covR* encoding His at position 66	This study
CovR-A81T	MGAS10870 with *covR* encoding Thr at position 81	This study
CovR-L155I	MGAS10870 with *covR* encoding Ile at position 155	This study
CovS-P285S	MGAS10870 with *covS* encoding Ser at position	This study

# derived from (14) for M3 strains and this study, Fig 1 for M1 strains

### CovS phosphatase deficient strains are hypovirulent in a skin/soft tissue infection model

**Fig 2:**
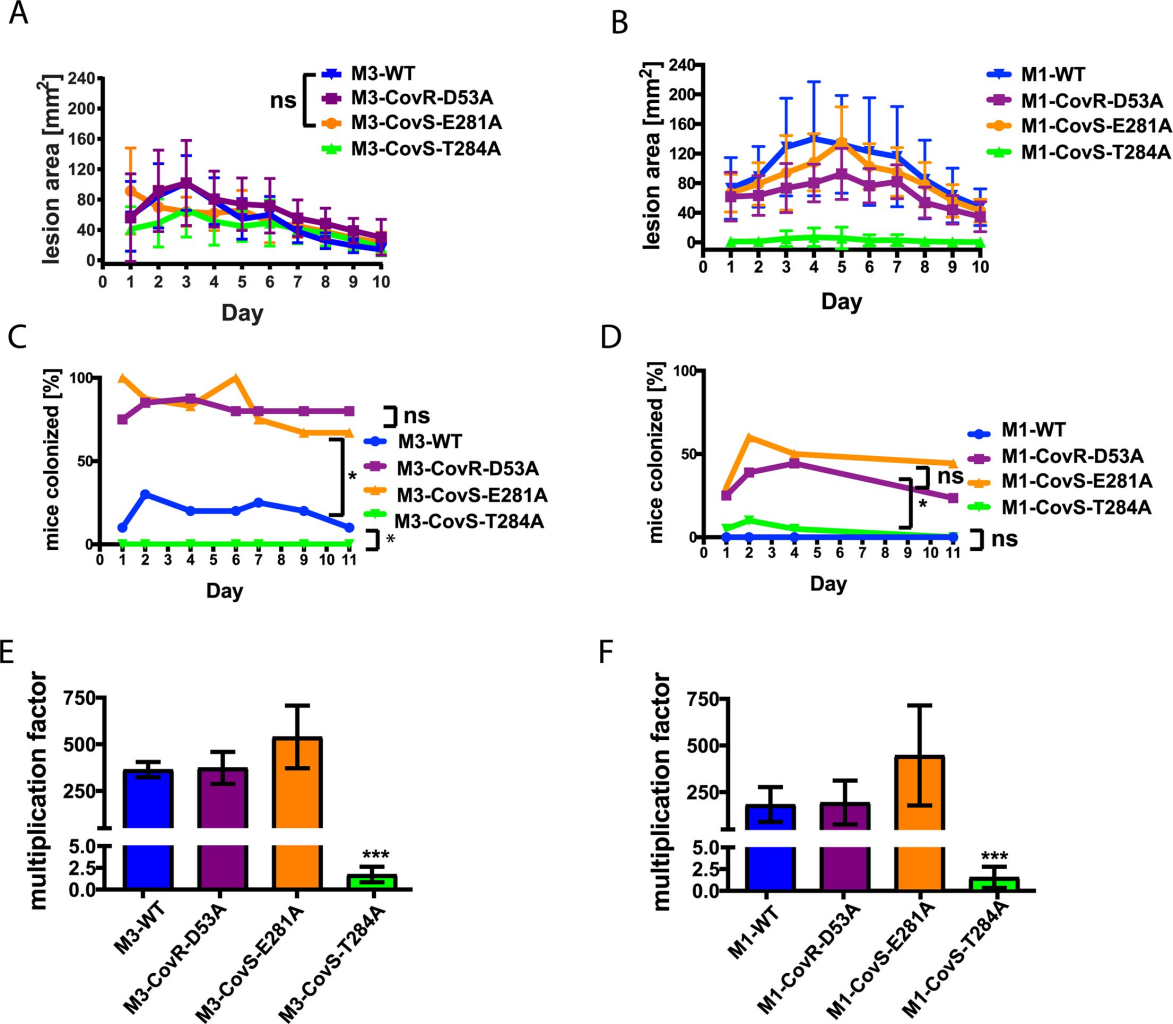
Influence of CovR~P levels on GAS virulence. (*A* and *B*) Lesion size measurements after mouse skin/soft tissue infection with 10^7^ CFU of indicated serotype M3 *(A)* and serotype M1 *(B)* GAS strains. 20 mice per strain were inoculated subcutaneously, and lesion areas were measured daily. Differences in lesion size between the GAS strains were statistically significant as measured by two-way ANOVA (P<0.05) with repeated measures unless otherwise noted; ns not significantly different. (*C* and *D*) Nasopharyngeal mouse model. Percentage (%) of mice colonized with the indicated serotype M3 *(C)* and serotype M1 *(D)* GAS strains on a given day. 20 mice per strain were inoculated via intranasal route with 1 x 10^8^ (M3) or 10^7^ (M1) CFU, respectively. Throat swabs were taken regularly, and recovered bacteria were plated on BSA plates to detect and enumerate β-hemolytic GAS colonies. Statistical comparisons were performed via a mixed linear model with repeated measures.; *, difference statistically significant (P<0.05). (*E* and *F*) Multiplication factors for indicated serotype M3 (*E*) and serotype M1 (*F*) GAS strains after 3h incubation in whole human blood. Data graphed are average and mean ± standard deviation of three biological replicates. One-way ANOVA was performed to assess if there was a difference among the strains with post-hoc comparisons performed using Tukey’s adjustment for multiple comparisons. *** *P*≤0.001.

To investigate the relationship between CovR~P levels and GAS virulence, we first employed a skin/soft tissue mouse model that mimics cellulitis, a common manifestation of GAS infection. For each strain, 20 mice were subcutaneously challenged with 10^7^ colony forming units (CFU) of GAS, and lesion size measurements were performed daily (Fig 2A and 2B). In the serotype M3 background (Fig 2A), all of the strains caused appreciable disease with strain M3-CovR-D53A causing the largest lesions over the course of the entire experiment (*P* <0.05 in comparison to each of the other three strains). The difference in lesion size generated by strain M3-CovS-E281A compared to the M3-WT strain was not significantly different (*P* = 0.99). The smallest lesions were produced by strain M3-CovS-T284A (*P* < 0.05 compared to each of the other three strains). However, it is notable that for some mice inoculated with strain M3-CovS-T284A, lesions started to appear more severe and showed ulceration after day 5 of the experiment, when healing of lesions had already set in for the other strains. We will address this finding later in the manuscript.

Among the M1 strains, the wild type strain was the most virulent with maximal average lesion area of ~150 mm^2^ on day 4 (*P* < 0.05 compared to the other three strains), followed by strain M1-CovS-E281A (*P* < 0.05 compared to strains M1-CovS-D53A and M1-T284A) (Fig 2B). Strain M1-CovS-T284A was the least virulent (Fig 2B) (*P* < 0.05 compared to the other strains). However, in contrast to its serotype M3 counterpart, only a few mice inoculated with M1-CovS-T284A evidenced any visible infection, and in these cases the lesions were small in size and without any sign of ulceration over the entire length of the experiment. Thus, we did not observe a consistent relationship between CovR~P levels and lesion size. Regardless, our results demonstrate that abrogation of CovS phosphatase activity in both GAS M1 and M3 serotypes attenuated virulence in the skin/soft tissue mouse model of infection.

### CovS phosphatase deficient GAS strains have decreased capacity to colonize the mouse oropharynx

Next, we investigated the effect of CovR~P levels on oropharyngeal GAS colonization following nasopharyngeal mouse challenge. For serotype M3 strains, 20 mice per strain were inoculated with 10^8^ CFU GAS in a 20 μl volume. The volume was reduced compared to previous studies to avoid aspiration of GAS into the lungs [36]. Unexpectedly, we observed a high death rate in mice inoculated with strains M3-CovS-E281A and M3-CovR-D53A, while no death occurred for mice inoculated with M3-WT or M3-T284A, albeit the same volume and CFU were used. For this reason, we subsequently reduced the inoculum to 10^7^ CFU for all serotype M1 strains. Despite the different inocula used for the M1 and M3 strains, we consistently observed that GAS strains with higher CovR~P colonized at lower rates (Fig 2C and 2D). Specifically, low CovR~P strains CovS-E281A and CovR-D53A colonized at significantly higher rates (~80% and 40–50% for serotype M3 and M1, respectively) compared to the wild type and CovS-T284A strains for both serotypes (*P* < 0.05 for all specified comparisons). For serotype M3, the wild type strain (medium CovR~P level) had a colonization rate intermediate to the low and high CovR~P strains (~30%, Fig 2C). For serotype M1, the wild type strain and M1-CovS-T284A (both high CovR~P) were rarely recovered shortly after initial inoculation (Fig 2C and 2D). Thus, we observed an inverse relationship between CovR~P levels and the capability of the GAS strains to colonize mouse pharyngeal tissue.

### CovS phosphatase activity contributes to GAS survival in human blood

To investigate whether changes in CovR~P levels affect GAS interaction with the human immune system, we performed a Lancefield bactericidal assay using heparinized whole human blood of three non-immune donors. GAS survival in whole human blood was determined by plating serial dilutions on BSA plates after 3 hours exposure of GAS cells to human blood, and multiplication factors compared to the inoculum were calculated for each strain (Fig 2E and 2F). Multiplication factors were generally lower for serotype M1 compared to serotype M3 (Fig 2E and 2F). This is consistent with the previous finding that an intact RocA protein negatively influences GAS survival in blood [39]. The multiplication factors between the respective wild type and CovR-D53A or CovS-E281A mutant strains were not significantly different (*P* > 0.05 for all comparisons). In stark contrast, CovS-T284A derivatives of both serotype M1 and M3 GAS completely lost the ability to survive and propagate in whole human blood (*P* < 0.001 for all comparisons) (Fig 2E and 2F). Thus, CovS phosphatase activity is crucial for GAS survival and propagation in whole human blood.

### LL-37 signaling influences CovS phosphatase activity

**Fig 3:**
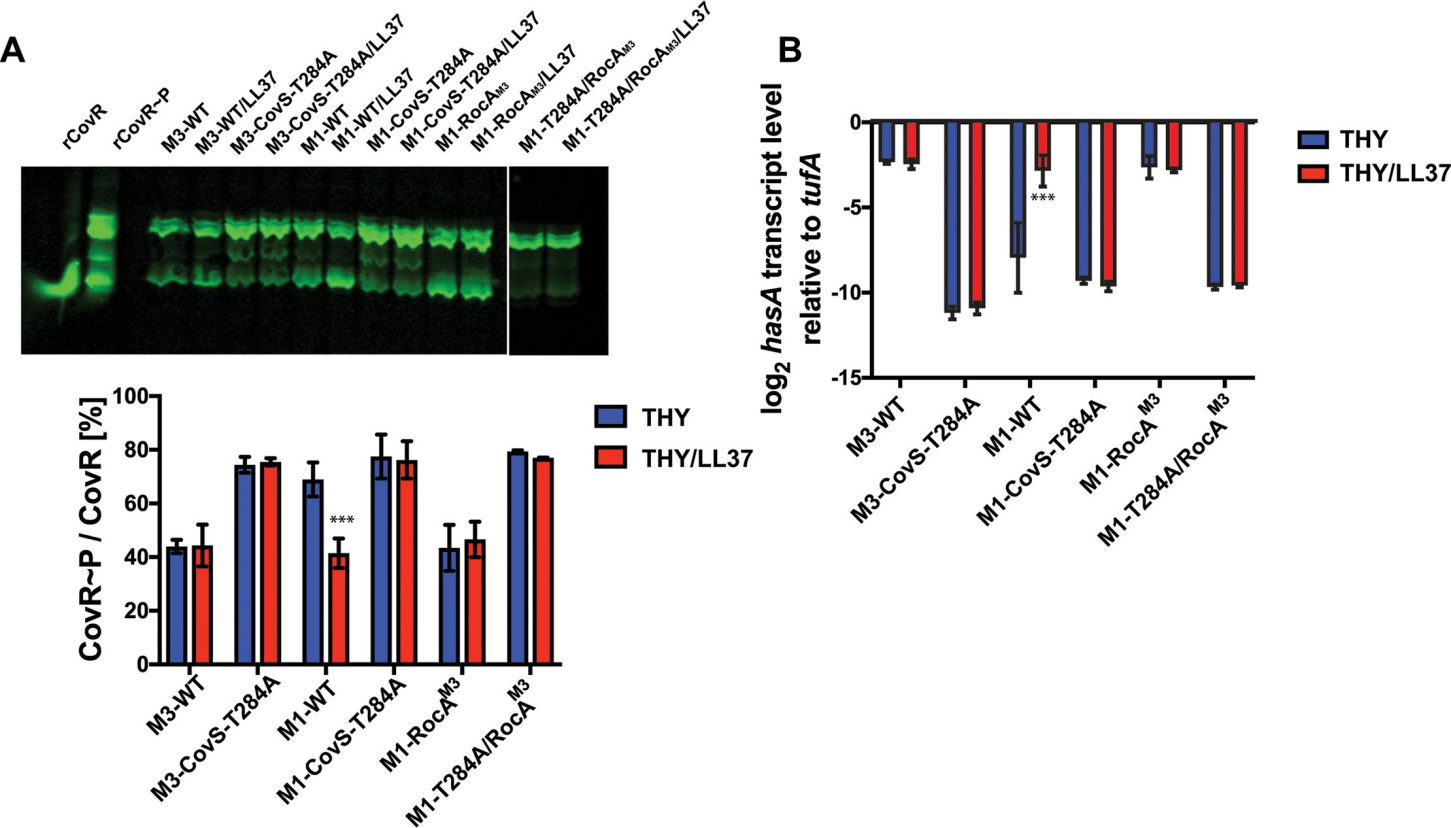
LL-37 signaling increases CovS phosphatase activity. (*A*) Representative Western blot (top) and bar representation (bottom) depicting CovR~P levels and (*B*) *hasA* transcript levels (means ± standard deviations, n = 4) of indicated GAS strains grown to mid-exponential phase in THY or THY supplemented with 100nM LL-37. *** denotes significant difference (*P*<0.001) between CovR~P or *hasA* transcript levels of indicated strain grown in THY and THY + LL-37 as determined using Student’s t-test using Bonferonni’s correction for multiple comparisons.

In standard laboratory medium CovR~P levels of strain M1-CovS-T284A are only slightly elevated compared to those in M1-WT, yet there were dramatic differences in virulence between the two strains in the skin/soft tissue infection model and bactericidal assay (Figs 1 and 2). One possible explanation for this discrepancy is that host factors increase CovS phosphatase activity during infection to decrease CovR~P levels and augment virulence factor production. Indeed, CovS senses the human antimicrobial peptide LL-37 and responds by lowering CovR~P levels [14, 18]. Thus, we next addressed the question whether LL-37 signaling is affected by the CovS-T284A mutation. To this end, we measured CovR~P and *hasA* (hyaluronic acid capsule) transcript levels in GAS strains grown in THY (standard laboratory medium) and THY supplemented with LL-37 (Fig 3A and 3B). Consistent with our previous findings [14], M1-WT CovR~P levels were reduced and consequently *hasA* transcript levels elevated in the presence of LL-37 compared to unsupplemented THY. In contrast, supplementation with LL-37 did not affect CovR~P or *hasA* transcript levels in strain M3-WT or strain M1-WT engineered to contain the truncated M3 version of RocA (M1-*rocA*^M3^) [39]. In both serotypes, the CovS-T284A strains had higher CovR~P and lower *hasA* transcript levels compared to the respective wild type strains when grown in unsupplemented THY. However, neither CovR~P nor *hasA* transcript levels were influenced by LL-37 in the CovS phosphatase deficient strains (Fig 3). Further, CovR~P and *hasA* transcript levels of CovS phosphatase deficient strains were not affected by an additional mutation in the *rocA* gene in either medium (see strain M1-CovS-T284A/*rocA*^M3^, Fig 3). We conclude that LL-37 increases CovS phosphatase activity in a GAS strain with an intact RocA.

### Impact of GAS serotype and CovR~P level on emergence of *covRS* mutations in different infection models

It is well established that strains with *covRS* mutations can emerge during human infection or animal passage, thereby giving rise to hypervirulent GAS isolates [23, 25–27]. To investigate how CovR~P levels in the context of GAS serotype influence the emergence of CovRS inactivated mutants, we amplified and sequenced the complete *covRS* operon from at least 25 randomly picked GAS colonies per strain isolated during each infection study (Table 2). In the skin/soft tissue model of infection, we recovered GAS colonies from skin lesions of five mice per strain on day 4. No additional mutations were found in colonies isolated from mice infected with strains M3-WT, M3-CovS-E281A, and M3-CovR-D53A. In contrast, a few of the colonies isolated from mice infected with strain M3-CovS-T284A had mutations in CovR, namely CovR-L155I and CovR-R66H. Since the lesions in some mice infected with M3-CovS-T284A looked more severe after day 5, we sequenced additional colonies isolated from these animals on day 10 (end point of experiment) and were able to detect colonies that had a duplication of *covS* nucleotides 100 to 131, which is predicted to result in a non-functional CovS due to frameshift (Table 2). Therefore, on rare occasions, it appears that mutations that abrogate CovS activity occurred late in the disease course for strain M3-CovS-T284A which likely accounted for the increase lesion size described above. As observed with the M3 strains, no GAS with additional *covRS* mutations were recovered from animals infected with strains M1-CovR-D53A and M1-CovS-E281A. Interestingly, however, unlike its serotype M3 counterpart, no additional mutations were found in GAS isolated from mice infected with strain M1-CovS-T284A. In contrast, a high number of colonies recovered from animals inoculated with the M1-WT isolate had mutations in the *covRS* systems as has been previously reported for this strain [25, 40] (Table 2). Many of the recovered strains had changes that truncated the CovS protein, whereas several colonies had non-synonymous SNPs in *covR* (A81T in CovR) or *covS* (R241S or P285S in CovS) (Table 2). No additional mutations were detected in any GAS strain of either serotype during nasopharyngeal mouse challenge or during growth in whole human blood. Thus, elimination of phosphatase activity by the CovS-T284A change abrogated the emergence of *covRS* mutations in the M1 background during skin/soft tissue infection but increased such emergence in the M3 strain, albeit primarily late in the infection course.

**Table 2:** Emergence of *covRS* mutations.

	subcutaneous challenge	nasopharyngeal challenge	growth in blood
**M3 serotype**			
CovR-D53A (0) ^§^	none	none	None
CovS-E281A (~20)	none	none	None
wild type (~40)	none	none	None
CovS-T284A (~70)	CovR-R66H, CovR-L155I, ^¶^duplication *covS*100-131	none	None
**M1 serotype**			
CovR-D53A (0)	none	none	None
CovS-E281A (~20)	none	none	None
wild type (~70)	*covS*1352 4bp del, *covS*1363 C/T stop, covS1441 G/T stop, CovS-P285S, CovS-R241S, CovR-A81T	nd	None
CovS-T284A (~75)	none	nd	None

§ CovR phosphorylation levels in %, derived from (14) for M3 and this study, Table 1 for M1

¶ found in skin lesions on day 10; nd not determined due to lack of colonization

### SNPs emerging during interaction with the host immune system can entail diverse effects on CovR function

**Fig 4:**
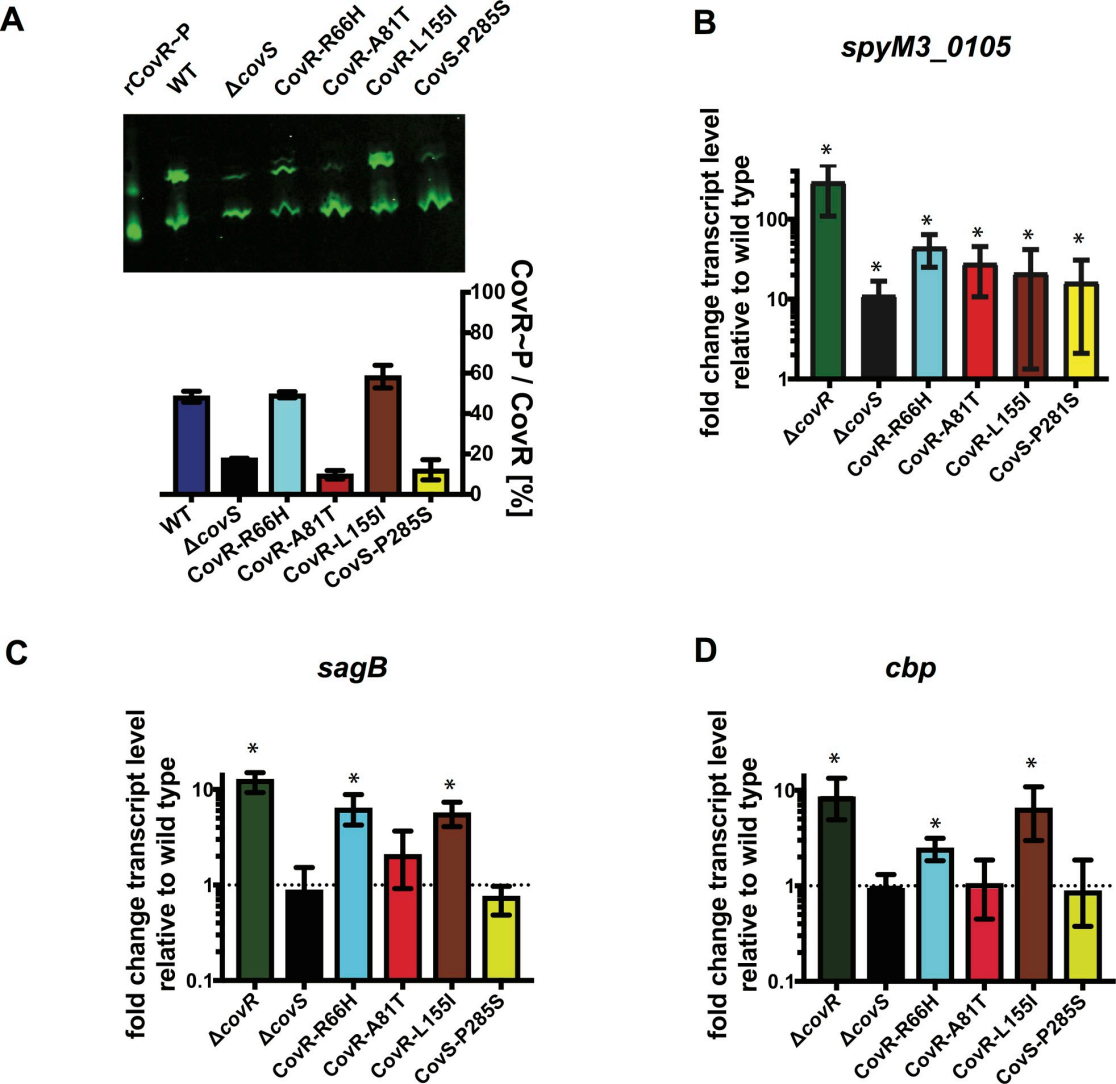
Effect of CovRS mutations selected during skin/soft tissue infection on CovR regulatory function. (*A*) CovR~P levels of indicated strains grown to mid-exponential phase in THY measured by Phostag-Western blot analysis (n = 2). *(B-D)* Transcript levels (means ± standard deviations; *n* = 4) of the indicated genes in the CovR/S-deleted or mutated strains relative to those of the wild type, as measured by TaqMan qRT-PCR. Strains were grown in THY to mid-exponential phase. * = *P* < 0.05 for gene transcript level in indicated strain relative to wild-type as determined by Student’s t-test using Bonferonni’s correction for multiple comparisons.

It has previously been shown that truncations in CovS mimic a *covS* deletion strain (e.g. reduced CovR~P levels), but much less is known about the consequences of non-synonymous single nucleotide polymorphisms (SNPs) in either *covR* or *covS* that arise during mouse challenge or human infection [38, 41]. Thus, we next sought to evaluate the effect of some of the previously uncharacterized SNPs isolated during our mouse infection study by generating the isoallelic GAS strains CovR-A81T, CovR-R66H, CovR-L155I, and CovS-P285A in the serotype M3 background (Fig 4). CovR~P levels in strains CovR-A81T and CovS-P285S were strongly reduced compared to the wild type and resembled that of a *covS* deletion strain. In contrast, strains CovR-R66H and CovR-L155I had CovR~P levels similar to the wild type (Fig 4A). SpeB is an actively secreted broad-spectrum protease whose production is abrogated by CovS inactivation [42, 43]. In accordance with the CovR~P levels, strains CovR-A81T and CovS-P285S had reduced SpeB activity on milk plates, while SpeB activity was not affected in CovR-R66H or CovR-L155I (S1 Fig). Next, we performed TaqMan qRT-PCR of various known CovR-regulated genes that have previously shown to be regulated by CovR via different mechanisms [44] to evaluate the effect of the mutations on CovR-mediated transcription regulation (Fig 4B–4D). Consistent with the CovR~P level analysis, in strains CovR-A81T and CovS-P285S transcript levels of *spyM3_0105*, *prtS*, *sagB*, and *cbp* (which encode a cell surface protein, an IL-8 degrading protease, a pore-forming toxin, and a pilus protein, respectively) resembled that of a *covS* deletion strain. Specifically, the transcript levels of *spyM3_0105* and *prtS* were >10 or ~2-fold elevated, respectively, compared to M3-WT (Fig 4B and S2 Fig), while transcript levels of *sagB* and *cbp* did not differ significantly compared to M3-WT (Fig 4C and 4D) (*P* > 0.05). Interestingly, despite CovR~P levels being similar to the wild type, repression of all genes studied was strongly relieved in strains CovR-R66H and CovR-L155I compared to the wild type (Fig 4B–4D) implying that functions aside from CovR phosphorylation (e.g. signal transduction, DNA binding) are affected in these mutants (*P* < 0.05 for all gene transcript levels).

### RNA sequencing reveals the basis for hypovirulent phenotype of CovS phosphatase deficient strains

**Fig 5:**
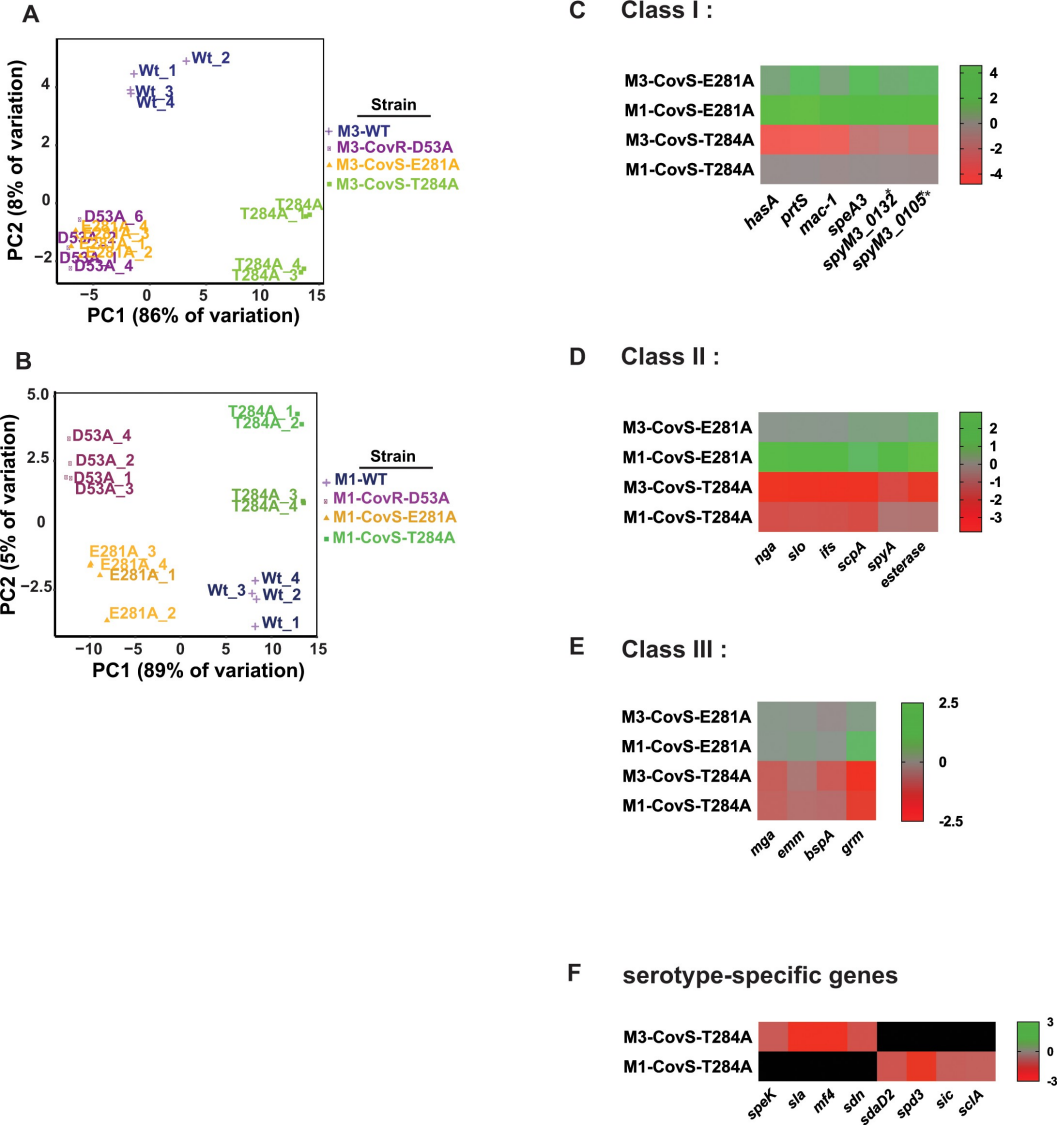
Transcriptome analysis of GAS strains with distinct CovR~P levels. Principle component analysis of RNAseq data derived from the indicated serotype M3 (*A*) and M1 (*B*) GAS strains grown in quadruplicate to late-exponential phase in THY. (*C-F*) Heat map of log_2_ transcript levels for selected CovRS-regulated genes in the indicated strains relative to their respective wild type. *spyM3_0132 is homolog to *5005_spy0142*, and **spyM3_0105 is homolog to *5005_spy0115*.

Next, we determined the transcriptomes of the eight strains used in the animal challenges to obtain mechanistic insights into the observed virulence differences. To this end, four biological replicates per strains were grown to late-logarithmic phase (OD = 0.9), and RNA was extracted and subjected to RNAseq analysis. Transcript levels were considered significantly different if the mean transcript level difference was ≥ 2.0 fold and the final adjusted P value ≤ 0.05 compared to the wild-type strain. By principle component analysis (PCA) biological replicates of each strain clustered together (Fig 5A and 5B). Consistent with the higher CovR~P levels in M1-WT compared to M3-WT, there were more genes differentially regulated compared to the wild type in strains M1-CovR-D53A and M1-CovS-E281A and less genes in M1-CovS-T284A than in the respective M3 serotype strains (Table 3). That is, the number of differentially regulated genes paralleled the differences in CovR~P between the wild type and the distinct isoallelic strains. In accordance with our virulence data, the transcriptomes of M3-CovR-D53A and M3-CovS-E281A were highly similar with 100 and 97 genes being differentially regulated compared to the wild type (Fig 5A, Table 3). Genes up-regulated compared to M3-WT included the known virulence genes *prtS* and *speA* (encoding a pyrogenic exotoxin). The transcriptomes of M1-CovR-D53A and M1-CovS-E281A were also similar. However, compared to their M3 counterparts, a larger number of known virulence factor encoding genes were up-regulated in these strains relative to M1-WT including the *has* operon, *nga* (NAD glycohydrolase), *slo* (streptolysin O), and *speC* (exotoxin). Moreover, transcript levels of several DNA binding proteins and genes involved in amino acid and sugar transport and metabolism were increased compared to the wild type.

**Table 3:** Number of genes differentially regulated compared to parental strains MGAS10870 (M3-WT) and MGAS2221 (M1-WT).

	Serotype M3	Serotype M1
D53A	100	171
E281A	97	139
T284A	65	25

In contrast, transcript levels of 41 and 23 genes were decreased in strains M3-CovS-T284A and M1-CovS-T284A compared to the wild type, respectively. Remarkably, genes further repressed in the CovS-T284A strains comprised those encoding nearly the complete repertoire of known GAS virulence factors ranging from secreted toxins to immune-modulating surface proteins (see Fig 5 and S2 Table), which likely explains the striking reduction in virulence of CovS phosphatase deficient GAS strains seen in our infection studies. Many genes with reduced transcript levels in the CovS-T284A strains have not been previously identified as part of the CovRS-regulon including *mga*, which encodes the multi-gene activator of numerous virulence genes, Mga, and part of its regulon, e.g. *emm* coding for M protein or *grm* (gene regulated by Mga) [45]. On the other hand, with the exception of the *dpp* operon (encoding a dipeptide permease) we did not identify genes involved in metabolism and transport being further repressed in the CovS-T284A strains, suggesting specific modulation of virulence gene expression in these strains. Thus, results of our transcriptome analyses can explain the hypervirulent phenotype of CovS phosphatase deficient strains by identifying (in part novel) CovR-mediated repression of a broad array of virulence factor encoding genes at high CovR~P levels.

### Transcription analyses reveal different promoter classes with distinct CovR~P promoter affinities

We further used our multi-strain, multi-serotype RNAseq data to differentiate three broad classes of CovR-regulated genes depending on their repression (T284A) and de-repression (E281A/D53A) profile (see examples in Fig 5C–5E). The first class (class I) encompasses genes that were de-repressed in the E281A and D53A strains in both serotype M1 and M3. These genes have been identified as part of the CovRS regulon by previous transcriptome studies [10, 17, 22, 25, 28, 38] and include well-known virulence genes like the *has* operon and *prtS*. Interestingly, these genes were only further repressed in the T284A strain compared to parent strain M3-WT while they were already fully repressed in M1-WT (Fig 5C). In contrast, we defined class II genes as those, whose transcript levels were affected in the E281A/D53A strains only in the M1-WT background but showed repression in the T284A strains for either serotype. These genes included *nga*/*slo* and *scpA* (C5 peptidase) (Fig 5D). As mentioned earlier, our transcriptome analysis identified novel virulence factor repression in both CovS-T284A strains. These genes, categorized as class III genes, were not increased in the CovS-E281A and CovR-D53A strains and have not previously been identified as part of the CovRS regulon in serotype M1 and serotype M3 GAS strains (Fig 5E). Additionally, transcript levels of several prophage-encoded, serotype-specific virulence factors like *speK* (exotoxin), *sla* (extracellular phospholipase) or *sdaD2*, *spd3*, and *sdn* (all secreted DNases) were reduced in the CovS-T284A mutant strains (Fig 5F). Due to their serotype-specific nature these genes could not be unambiguously assigned to one of the described major classes. Extracellular DNases have been shown to contribute to GAS pathogenesis [46]. To gain insight into the physiological consequences of our transcription data, we performed DNase activity tests and found that indeed DNase activity was significantly reduced in supernatants derived from CovS-T284A cells compared to that of the respective parental strains (S3 Fig).

**Fig 6:**
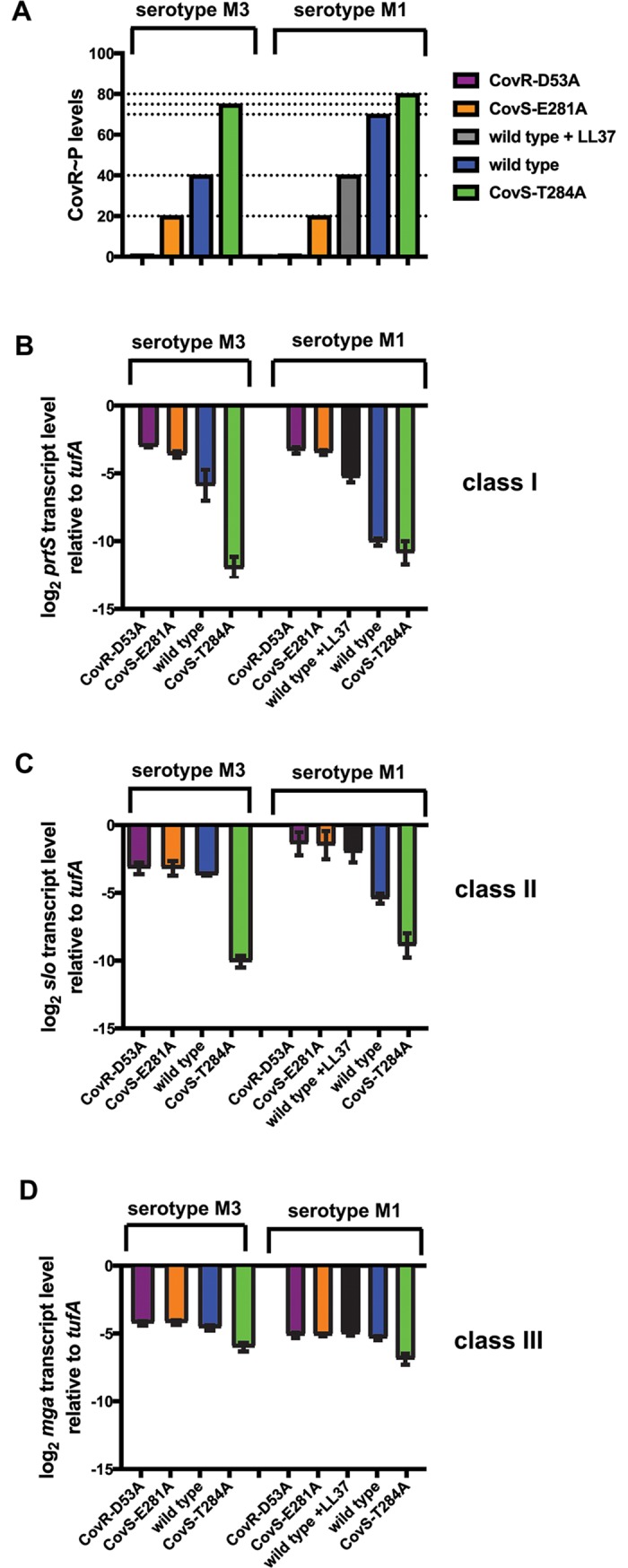
Influence of CovR~P on transcript levels of genes representing distinct classes. (*A*) Schematic depiction of CovR~P levels in the indicated GAS strains. Values were derived from previous Phostag-Western blot analyses of CovR~P status. (*B-D*) Transcript levels (means ± standard deviations; *n* = 4) of indicated genes that are representative for distinct gene classes in the isoallelic GAS strains relative to those of the wild type, as measured by TaqMan qRT-PCR. Strains were grown in THY to late-exponential phase.

Next, we employed TaqMan qRT-PCR to confirm the transcript level pattern of selected genes exemplifying the different classes of CovR-regulated genes revealed by RNAseq (Fig 6 and S4 Fig). To enable a better comparison of transcript levels vs. CovR~P in both serotypes, we also included strain M1-WT grown in THY supplemented with 100nM LL-37 (CovR~P is ~40% as for M3-WT) in our analyses. The qRT-PCR results were in concert with the RNAseq data. By contrasting the level of CovR~P with the degree of transcriptional regulation we were able to deduce differences in CovR~P dependency for the regulation of distinct promoter classes. The transcript levels of class I genes, exemplified by *prtS* (Fig 6B) and *hasA* (S4B Fig), revealed differential regulation over a range of 20–70% CovR~P, above which no further repression was detectable. In contrast, gene regulation of class II genes, such as *slo* (Fig 6C) and *scpA* (S4C Fig), was not affected by varying CovR~P below a level of 40%, but was dramatically impacted when CovR~P was increased from 40% to beyond 70%. *Mga* (Fig 6D) and *emm* (S4D Fig), as examples for class III genes, showed the highest CovR~P dependency with CovR-mediated repression only observed at CovR~P above 70%.

### CovR binds to sequences in the *mga* promoter region

**Fig 7:**
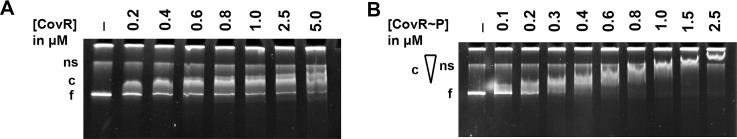
CovR binding to *mga* promoter DNA. Electromobility shift assay (EMSA) depicting binding of unphosphorylated (*A*) and *in vitro* phosphorylated (*B*) recombinant CovR to DNA of the serotype M3 GAS *mga* promoter. DNA was incubated with increasing amounts of protein as indicated and samples were separated by electrophoresis on a 5.5% native PAA-gel at 100V over 2h. Gels were stained with ethidium-bromide. Ns, non-specific; c, protein-DNA complex; f, free DNA. The triangle symbolizes increasing molecular weight of the complex as indicated by stronger retardation.

Mga and its regulated genes have previously not been identified as part of the CovR regulon in serotype M1 or M3 GAS [17, 25]. Our transcriptome data, however, showed that the expression of *mga* and several Mga-controlled genes such as *emm* or *grm* were down-regulated in M3-CovS-T284A and M1-CovS-T284A compared to the respective parental strain (Fig 5E and Fig 6D). Given the presence of potential CovR-binding sites (ATTARA) within the *mga* promoter, we next performed electrophoretic shift mobility analyses (EMSA) to address the question whether CovR binds to the promoter of *mga* and whether this binding is dependent on the phosphorylation status of the protein. A PCR fragment of ~500bp encompassing the *mga* promoter amplified from M1-WT genomic DNA (DNA sequences of *mga* promoter regions from M1-WT and M3-WT have 94% nucleotide identity) was incubated with increasing concentration of unphosphorylated or *in vitro* phosphorylated purified CovR protein, and samples were separated on a TBE-PAA gel. Although unphosphorylated CovR was able to bind the *mga* promoter DNA to create a low molecular weight complex, increasing protein concentrations up to 5 μM did not appreciably change the binding behavior (Fig 7A). By contrast, increasing concentrations of CovR~P progressively resulted in complexes of higher molecular weight as typically seen in CovR/CovR~P promoter binding assays of genes known to be directly regulated by CovR [22, 28] (Fig 7B). In accordance with our transcription data, high concentrations of CovR~P were needed for effective binding of the *mga* promoter.

## Discussion

Although phosphorylation of response regulator proteins is critical for bacterial pathogenesis, there remains limited understanding of how variation in response regulator phosphorylation impacts bacterial virulence at diverse infection sites. Herein we employed transcriptome and virulence assays of an array of isoallelic serotype M1 and M3 GAS strains to assess the impact of multiple, distinct phosphorylation levels of the key response regulator CovR on GAS pathophysiology.

For our virulence assays, we chose the mouse models of skin/soft tissue infection and nasopharyngeal colonization. Previous studies on the contribution of CovRS to GAS infection using these models have come to varying conclusions [20, 25, 26, 35, 36], which may be explained by serotype-specific differences, the use of strains with diverse inactivated regulators with potentially additional functions (Δ*covS* vs. Δ*rocA*) or possible emergence of hyper-virulent clones during infection. In contrast, all studies to date using the intraperitoneal (i.e. bacteremia) model had consistently found increased GAS virulence with decreasing CovR~P levels [5, 21, 22, 25, 34]. Confirming many previous observations, our assays indicate both serotype- and site-specific effects on the impact of CovRS inactivation on GAS pathogenesis. Similar to results obtained by Dalton *et al*. [35], we saw an inverse correlation between CovR~P levels and virulence of our M3 strains in the skin/soft tissue model. This result was not mirrored by our M1 strains. However, the hypervirulent phenotype of M1-WT in our mouse skin/soft tissue model likely stems from the emergence of CovS inactivating, SpeB^-^ mutations early in the infection course. It has been shown that a mixture of wild type and *covS*-inactivated M1 GAS produces larger skin lesions compared to a *covS* deletion strain [26], consistent with skin-specific increase in virulence of wild-type vs. *covS*-inactivated M1 GAS described by Sumby *et al*. [25]. Although inactivating CovS is believed to reduce GAS fitness in the upper respiratory tract [36, 47], we observed a profound increase in nasopharyngeal colonization rates for strains with lower CovR~P levels for both serotypes. Despite the limitations of the nasopharyngeal mouse model, a similar study using an M18 strain found that increasing CovR~P levels by repairing a naturally occurring RocA mutation also decreased colonization rates [20]. While previous studies solely analyzed the effect of lowering CovR~P on GAS infectivity, the most striking conclusion of our virulence data was the consistent hypovirulent phenotype of the CovS-T284A strains. In both M1 and M3 GAS serotype, these CovS phosphatase deficient strains caused the smallest lesions in the skin/soft tissue model, had a strongly reduced capacity to colonize the mouse oropharynx and did not survive neutrophil killing in whole human blood. Thus, CovS phosphatase activity strongly influences GAS overall ability to cause disease.

Another key finding from our animal studies was the effect of CovR~P levels on emergence of GAS strains with CovRS inactivating mutations. Consistent with the concept that the primary selective pressure for CovRS mutations is to decrease CovR~P levels, we only observed mutations in strains whose initial CovR~P levels were ≥ 70%. The absence of additional mutations in the E281A strains suggest that there is no selection pressure to reduce CovR~P beyond ~20% and is in accordance with human data that GAS strains with CovR inactivating mutations are rare compared to CovS. Surprisingly, increasing CovR~P levels via the T284A mutations did not evoke high levels of CovRS mutations. The latter occurred late in the infection course in a very limited number of animals infected with M3-CovS-T284A and not at all in mice infected with M1-CovS-T284A. Given the small lesion sizes, we speculate that the profound hypovirulence induced by the T284A mutation inhibited such emergence.

Our transcriptome analysis offers an explanation for the hypovirulent phenotype by revealing specific down-regulation of nearly the entire repertoire of virulence factor encoding genes in the CovS-T284A strains. Interestingly, plasmid-derived overexpression of RocA in M1-WT produced a similar virulence gene repression profile [39]. Given our finding that RocA impairs CovS phosphatase activity (Fig 3), we speculate that RocA overexpression results in CovR~P levels similar to the CovS-T284A strains. Among the identified novel CovR-repressed virulence genes in the CovS-T284A strains were *mga* and Mga-regulated genes. Mga is a well-known activator of numerous GAS virulence factors, such as M protein, and is thought to be particularly important in the early stages of infection [45, 48]. Thus it is likely that decreased *mga* expression played a pivotal role in the observed hypovirulence of our CovS phosphatase deficient strains. In addition, down-regulation of the Mga regulon has been shown to prevent *in vivo* selection of hypervirulent SpeB negative *covRS* variants [32], and thus the low *mga* and *emm* transcript levels in the CovS-T284A strains may have negatively affected hypervirulent isolate emergence. Although an indirect relationship between CovR and Mga is possible [49], our DNA binding analysis suggests that CovR could directly regulate *mga*. In accordance with this, we found several potential CovR-binding sites (ATTARA) within the *mga* promoter region, in particular an ATTARA sequence directly upstream of the P2 and downstream of the P1 promoter [50] as well as one partially overlapping a CodY binding site [51]. Nonetheless, in serotype M1 and M3 GAS, a functional protein-DNA complex seems to be only achieved at high CovR~P levels as seen in the CovS phosphatase deficient strains.

Our multi-strain, multi-serotype approach during our transcription level analysis allowed us to distinguish three distinct classes of CovR-regulated genes on the basis of their CovR~P dependency for gene regulation. In addition, we have previously described a group of CovR-regulated genes (e.g. *sagB*, *cbp*, *covR*), whose transcription regulation is independent of CovS [14, 44]. Hence, for this group, which we designate as class 0 in this context, CovR~P of only 20% is sufficient to repress gene expression. Together our data suggests classes of promoters repressed under distinct CovR~P concentrations increasing from class 0 to class III. This CovR~P dependency is likely determined by a combination of different affinities for CovR binding sites (as suggested by Jain *et al*. [39]) and diverse DNA-binding mechanisms (cooperativity, CovR oligomerization state, or interaction with other regulators or RNA polymerase) [6, 12, 44, 52–55] and requires further investigations. Regardless, the gradual repression of promoter groups establishes the basis for coordinated expression of GAS virulence factors in response to changing environmental cues.

Adjusting gene expression in adaptation to environmental niches is pivotal for pathogenic bacteria. Recently, this function has been increasingly attributed to the phosphatase activity of bi-functional HisKA-family kinases [56–58]. Thus, besides limiting crosstalk between homologous TCS [59], histidine kinase phosphatase activity evidently fulfills an important role in sensing extracellular signals. The currently established environmental signals that modulate the function of CovRS TCS likewise seem to target CovS phosphatase rather than kinase activity. Previous investigations revealed that inactivation of CovR by CovS is required for survival of GAS under stressful conditions such as the presence of LL-37, iron starvation or acidic stress suggesting that stress signals activate CovS phosphatase activity in M3 or M6 strains [60–62]. Mg^2+^ reduces CovS phosphatase activity by an unknown mechanism thereby increasing intracellular CovR~P [14]. Herein we show that the presence of accessory protein RocA in MGAS2221 also diminishes CovS phosphatase activity. We speculate that RocA forms a hetero-oligomeric complex with CovS thereby stabilizing a CovS phosphatase incompetent conformation (activated state) [63]. Further, we show that the antimicrobial peptide LL-37 in turn increases CovS phosphatase activity. LL-37 has been demonstrated to bind directly to CovS [64] but its effect on CovR~P and transcription regulation is only observed in GAS strains expressing a functional RocA protein. Thus, we hypothesize that LL-37 increases CovS phosphatase activity indirectly by displacing RocA from the hetero-oligomeric complex to allow formation of a CovS phosphatase competent conformation. The antagonism between Stk mediated phosphorylation of CovR T65 and CovS phosphorylation at D53 adds an additional layer of complexity to regulation of CovR function [22]. The multi-faceted regulation of CovS phosphatase activity highlights its crucial function in adjusting CovR~P status and thus the expression of CovR-controlled virulence genes.

Bacterial TCSs have been proposed as potential therapeutic targets [65–71]. General inhibition of CovS function is unlikely to be desirable in GAS given the hypervirulence of Δ*covS* strains. However, the data presented in this study suggests that specifically targeting CovS phosphatase activity might be promising. CovS is present in all GAS serotypes, and abolishing CovS phosphatase activity markedly reduced GAS virulence in all three infection models. Notably, hypovirulence was even detected in serotype M1 GAS, a strain with low intrinsic CovS phosphatase activity and therefore high baseline CovR~P. Further, phosphatase activity of other bifunctional HisKA-family histidine kinases has been shown to play an important role in regulating infectivity of both Gram-positive and Gram-negative pathogens. For example, Liu *et al*. demonstrated that a T247A mutation (homolog to CovS-T284A) of *Salmonella enterica* EnvZ, mimicking a conserved pH-controlled mechanism of HK phosphatase activity ablation, increased macrophage infectivity due to accumulation of OmpR~P and downstream activation of *ssrA-ssrB* genes [58]. Mutations of WalK PAS domain that modulate WalK phosphatase activity also attenuated virulence of *S*. *pneumoniae* in a murine infection model [72]. The Spinola group has suggested the use of phosphatase inhibitors towards histidine kinase CpxA to treat certain urinary tract infections caused by uropathogenic *E*. *coli* [73, 74]. These examples corroborate the importance of HK phosphatase activity in bacterial virulence and suggest a broader application of this approach with regards to other histidine kinases in pathogen bacteria.

Together our study provides novel insights into mechanisms of GAS virulence factor regulation and establishes an important role of CovS phosphatase activity in controlling GAS pathogenicity.

## Materials and methods

### Ethics statement

This study was carried out in strict accordance with the recommendations in the Guide for the Care and Use of Laboratory Animals of the National Institutes of Health. Protocol #00001455-RN00 was approved by The University of Texas MD Anderson Cancer Center Institutional Animal Care and Use Committee. All efforts were made to minimize suffering. Human blood samples were drawn and used under IRB protocol #0110–0015 approved by Houston Methodist Research Institute Review Board. Written informed consent was obtained from all donors.

### Bacterial strains and media

All GAS strains used in this study are derivatives of either strain MGAS10870 (serotype M3, herein called M3-WT) or strain MGAS2221 (serotype M1, herein called M1-WT), two clinical isolates that are known to have wild type *covRS* sequence (see Table 1). Primers for strain creation are listed in S1 Table. Single nucleotide exchanges were introduced into chromosomal DNA of strain M1-WT or M3-WT via homologues recombination using the integrative plasmids pBB740 or pJL1055, respectively, as described in [28] to create isoallelic strains that differ only by the presence of a single amino acid replacement in CovR or CovS. GAS strains were statically grown in Todd Hewitt broth supplemented with 0.2% yeast (THY) at 37°C and 5% CO_2_. When appropriate, chloramphenicol was added to 5μg/ml. Bacteria were plated on tryptic soy agar with 5% sheep blood (BSA) plates.

### Detection of CovR phosphorylation levels *in vivo*

Recombinant CovR was purified and phosphorylated as described [28] and served as control. GAS lysates were prepared and separated on 12.5% SuperSeq Phostag gels (Wako, USA), and un/phosphorylated CovR species were detected using a polyclonal anti-CovR antibody and the Odyssey imaging system as described previously [14]. Independent Western blots were repeated at least twice.

### Mouse challenge

Strains were grown in 200 ml THY to mid-exponential phase and harvested by centrifugation at 9000 rpm. Cell pellets were washed twice with ice-cold PBS buffer, re-suspended in 4 ml PBS/20% glycerol solution and stored in aliquots at -80° C until use. CFU counts for each strain were determined by plating dilutions of the samples on BSA plates at least three times and confirmed after mice inoculation. All animal experiments were performed in a blinded fashion.

#### Skin/Soft tissue challenge

To mimic cellulitis, 20 immunocompetent, hairless mice (Crl:SKH1-Elite) (Charles River Laboratories) were inoculated subcutaneously with 1 x 10^7^ CFU per strain. This dose was optimized for M3-WT and M1-WT strains to result in lesion formation and ulceration without inducing near mortality. Lesion area (in mm^2^) was measured daily until day 10, when most lesions were clearly healing. Lesion size comparisons were performed via two-way ANOVA repeated measures analysis with Tukey’s correction for multiple comparisons. Additionally, a mixed linear model with repeated measures was also performed on the data with the same results. On day 4, five mice per strain were euthanized and skin lesions excised. Cellular material was ground in 4 mL PBS using an electric grinder, and dilutions in PBS were plated on BSA agar for subsequent testing for *covRS* mutations.

#### Nasopharyngeal challenge

To monitor nasopharyngeal colonization of GAS, 20 CD-1 IGS mice (Charles River laboratories) per strain were inoculated with 1 x 10^7^ (M1) or 1 x 10^8^ CFU GAS in 20μl PBS. The volume was lowered compared to previous studies to avoid aspiration of GAS into the lungs. Over a period of 11 days, the mice nasopharyngeal tissue was swabbed, bacteria recovered in 300μl PBS and plated on BSA plates for enumeration of β-hemolytic colonies. A mixed linear model with repeated measures was used to determine differences in percent of mice colonized over time with Tukey’s correction for multiple comparisons.

### Lancefield bactericidal assay

Whole blood was drawn in sodium heparin tubes (Becton Dickinson) from three consented, healthy, non-immune donors under IRB protocol #0110–0015. GAS growth in blood was performed as described in [21]. Indicated strains were grown in THY as described for animal experiments. 20–100 CFU of each GAS strain was inoculated in 300μl human blood containing 10% THY, respectively. Samples were incubated for 3h at 37°C in 5% CO_2_ with end-to-end rotation. CFU/ml were determined by plating serial dilutions in PBS on BSA plates for enumeration of β-hemolytic colonies. Multiplication factors were determined by dividing CFU/ml after 3h incubation by CFU/ml in the inoculum. The experiment was performed in triple biologic replicates on two separate occasions. Data were analyzed using one-way ANOVA followed by post-hoc analysis using Tukey’s correction for multiple comparisons.

### DNase activity assay

The DNase activity in filtered culture supernatants from M1 and M3 wild type and CovS-T284A strains grown to late-exponential phase (OD = 0.95) was assayed. To this end, 100ng PCR-derived GAS DNA was incubated for 20 min at 37°C in 1x NEB 2 buffer (New England Biolabs) with 0.5μl of the respective supernatant. The remaining DNA was quantified using Quant-IT Pico Green dsDNA reagent (Thermo Fisher Scientific) according to the manufacturer’s instructions. Fluorescence was detected using a fluorescence microplate reader with 480 nm excitation and 520 nm emission wavelength. DNA concentrations were calculated using a standard curve with known DNA concentrations. Three biological replicates were assayed on two separate occasions (n = 6).

### Electrophoretic shift assay (EMSA)

The ~500bp encompassing *mga* promoter region was amplified from M1-WT genomic DNA by PCR using primers listed in S1 Table. The *mga* promoter region from M1-WT and M3-WT share 94% sequence identity, such that results from our EMSAs using serotype M1 DNA are likely applicable to serotype M3. Purified PCR product was incubated in TBE-buffer with the indicated amount of CovR or CovR phosphorylated with acetyl phosphate at 37°C for 15 min as described [28]. Subsequently, samples were separated on a 5.5% TBE-PAA gel for 2h at 120V and stained with ethidium bromide.

### Emergence of *covRS* mutations

Per strain and experiment, at least 25 colonies were picked, and the complete *covRS* operon was PCR amplified and sequenced using the primers listed in S1 Table. Sequences were analyzed with Sequencher 5.4.6 using the *covRS* sequence of M3-WT or M1-WT, respectively, as template.

### RNA sequencing (RNA seq)

Strains were grown in quadruplicate to late-exponential phase in THY. RNA was isolated using the RNeasy minikit (Qiagen). RNA sequencing was performed at the MD Anderson Sequencing and Microarray Facility, and data analysis was performed as described using the M3-WT and MGAS5005 (an M1 strain) genome, respectively [22]. A total of 88 out of 1853 (4.7%) genes (M3 serotype) and 73 out of 1849 (4%) genes (M1 serotype) were excluded from the analysis due to low expression levels. Transcript levels were considered significantly different between the isoallelic and wild-type strains if the mean transcript level difference was ≥ 2.0 fold and the final adjusted P value was ≤ 0.05. Transcriptome data have been deposited in the GEO database under accession number GSE121313.

### TaqMan qRT-PCR

Strains were grown as described for RNA seq. Approximately 300 ng RNA per sample was converted to cDNA using a high-capacity reverse transcription kit (Applied Biosystems). TaqMan quantitative real-time PCR (qRT-PCR) was performed on an Applied Biosystems Step-One Plus system as described [22] using primers and probes listed in S1 Table. Two biological replicates were performed on two separate occasions (n = 4). Transcript levels between wild type and derivative strains were compared using a two-sample *t* test (unequal variance) with a P value of ≤0.05 following Bonferroni’s adjustment for multiple comparison and a mean transcript level of ≥ 2.0-fold change being considered as statistically significant different.

## Supporting information

S1 Table Plasmids, primers and probes. (DOCX)

S2 Table Selected genes differentially regulated in the indicated strains compared to respective wild type strain. (DOCX)

S1 Fig Milk plate assay. SpeB protease activity was assessed by the size of a clear zone around the bacterial growth on casein milk agar plates. Strains shown clockwise are WT = MGAS10870 (M3-WT), R66H = M3-CovR-R66H, L155I = M3-CovR-L155I, A81T = M3-CovR-A81T, P285S = M3-CovS-P285S, and ΔS = M3Δ*covS*.
(TIF)

S2 Fig PrtS gene transcript level in various CovR/S isoallelic strains. Transcript levels (means ± standard deviations; n = 4) of *prtS* in the CovR/S-deleted or mutated strains relative to those of the wild type, as measured by TaqMan qRT-PCR. Strains were grown in THY to mid-exponential phase. * = *P* < 0.05 for gene transcript level in indicated strain relative to wild-type as determined by Student’s t-test using Bonferonni’s correction for multiple comparisons.
(EPS)

S3 Fig DNase activity of wild-type and CovS-T284A derivatives. DNase activity of culture supernatants was determined as described in the Materials and Methods. Shown is mean ± standard deviation of residual DNA following incubation with filtered supernatant of indicated strains. Experiments were performed in triplicate on two separate days. * = *P* < 0.05 for residual DNA level in indicated CovS-T284A strain relative to wild-type as determined by Student’s t-test using Bonferonni’s correction for multiple comparisons.
(EPS)

S4 Fig Influence of CovR~P on transcript levels of genes representing distinct classes. (*A*) Schematic depiction of CovR~P levels in the indicated GAS strains. Values were derived from previous Phostag-Western blot analyses of CovR~P status. (*B-D*) Transcript levels (means ± standard deviations; *n* = 4) of indicated genes that are representative for distinct gene classes in the isoallelic GAS strains relative to those of the wild type, as measured by TaqMan qRT-PCR. Strains were grown in THY to late-exponential phase.
(TIF)
