# Supplementary material for: Phosphatase activity of the control of virulence sensor kinase CovS is critical for the pathogenesis of group A streptococcus
Source: PLoS Pathog. 2018 Oct 31;14(10):e1007354. doi: 10.1371/journal.ppat.1007354 (PMC6231683; doi:10.1371/journal.ppat.1007354)
Supplement: S1 Table — (DOCX) [file ppat.1007354.s001.docx]

**Table S1 Plasmids, primers and probes**

| **Plasmid** | **Description** | **Reference** |
| --- | --- | --- |
| pJL1055 | Integrative, temperature sensitive vector used for M3 GAS, cm^R^ | Li J, 1997 |
| pJL-CovR_100_ | Part of *covRS* sequence cloned into pJL1055 via *BamHI* and *HindIII* | This study |
| pBB740 | Integrative vector used for M1 GAS, cm^R^ |  |
| pBB740-*covS* | *covS* sequence cloned into *BamHI* site of pBB740 | This study |
| pBB740-*rocA*^M3^ | Serotype M3 *rocA* sequence with flanking serotype M1 sequence | This study |
| pTYB1-*covR* | CovR overexpression vector | Horstmann, 2011 |
| **Primer** | **Sequence (5’ to 3’)** | **Purpose** |
| CovRS5’ | GAC CAT AGA GGG CAG AGA | 5’ primer for amplifying *covS* gene region and for sequencing |
| CovRS3’ | CAG TTG TGG CAA AAG ACT GC | 5’ primer for amplifying *covS* gene region and for sequencing |
| Seq3 | ATC ACT GTC ATC TTT ACC CC | *covRS* sequencing primer |
| pJLCovR_100__fwd | AGCAGGATCCGTATTTTGACCATAGAGGGC | pJL-CovR_100_ cloning |
| pJLCovR_100__rev | GACTAAGCTTGTAAAATTAGAGTCCACCTC | pJL-CovR_100_ cloning |
| covR-R66H_fwd | GGT TTT GAA GTG ACC CAT CGT TTG CAA ACC GAA | Quick-change mutagenesis |
| covR-R66H_rev | TTC GGT TTG CAA ACG ATG GGT CAC TTC AAA ACC | Quick-change mutagenesis |
| covR-A81T_fwd | C ATG ATG ATG ACT ACG CGT GAT TCT A | Quick-change mutagenesis |
| covR-A81T_rev | T AGA ATC ACG CGT AGT CAT CAT CAT G | Quick-change mutagenesis |
| covR-L155I_fwd | GAC GAT GAG ATT TCT ATC ACT AAA CGT GAA TAT G | Quick-change mutagenesis |
| covR-L155I_rev | C ATA TTC ACG TTT AGT GAT AGA AAT CTC ATC GTC | Quick-change mutagenesis |
| covS-P285S_fwd | CAT GAA TTA CGA ACA TCG GTT GCG ATT ATT A | Quick-change mutagenesis |
| covS-P285S_fwd | T AAT AAT CGC AAC CGA TGT TCG TAA TTC ATG | Quick-change mutagenesis |
| covR-D53A-5’ | GAT TTA ATC CTG CTT GCC TTA ATG TTA CCA GAG | Quick-change mutagenesis |
| covR- D53A-3’ | CTC TGG TAA CAT TAA GGC AAG CAG GAT TAA ATC | Quick-change mutagenesis |
| covS-E281A-5’ | tag tga tgt cag cca tgc att acg aac acc ggt tg | Quick-change mutagenesis |
| covS-E281A-3’ | caa ccg gtg ttc gta atg cat ggc tga cat cac ta | Quick-change mutagenesis |
| covS-T284A-5’ | CAG CCA TGA ATT ACG AGC ACC GGT TGC GAT TAT TAA | Quick-change mutagenesis |
| covS-T284A-3’ | TTA ATA ATC GCA ACC GGT GCT CGT AAT TCA TGG CTG | Quick-change mutagenesis |
| 5’ mgap_M3 | GCC AAC GAA AAA AGA CAA GT | EMSA |
| 3’ mgap_M3 | GAC TTG TAA ACA ACT TAC TTA C | EMSA |
| **qRT primer and probes** |  |  |
| 5’- *hasA* | CCG CGA CAA TGG TCA AAT TA | TaqMan primer |
| 3’- *hasA* | CGT CAG CGT CAG ATC TTT CAA A | TaqMan primer |
| *hasA* | GGT ATC CGT GGG CTC AGT CAC ACC A | TaqMan probe |
| 5’- *prtS* | AAG GAG CTT GGG ACA AGG GAT A | TaqMan primer |
| 3’- *prtS* | TGA TGG GCC GGA TCG A | TaqMan primer |
| *prtS* | CAA TAA CTG CGA CAA CCT TGC CTT GTC CT | TaqMan probe |
| 5’- *sagA* | TTG CTC CTG GAG GCT GCT | TaqMan primer |
| 3’- *sagA* | CTT CCG CTA CCA CCT TGA GAA T | TaqMan primer |
| *sagA* | ACC ACT TCC AGT AGC AAT TGA GAA GCA ACA AG | TaqMan probe |
| 5’- *cbp* | TTT AAG TGC TGG TAT GTT CCG GAT A | TaqMan primer |
| 3’- *cbp* | CCT TCC GCA TAT TTT CTG ATA ATG A | TaqMan primer |
| *cbp* | CCC CCA AAA CCA GGA GAA GAG CCT C | TaqMan probe |
| 5’- *spyM3_0105* | TTA CAA ACT AAA AAA CGA CGG GAA A | TaqMan primer |
| 3’- *spyM3_0105* | ATT CCA TTT ATC TTT GTA ACC TTC TTC AAG | TaqMan primer |
| *spyM3_0105* | AGA CAC CTA CGG CCG TCA ATC ACA CAC | TaqMan probe |
| 5’- *slo* | GAA ATA TCC GTA TCA TGG CTA GAG AGT | TaqMan primer |
| 3’- *slo* | CAC ATC TCT TTC GTC GAT CAC TTT | TaqMan primer |
| *slo* | GCC ACC ATT CCC AAG CTA AGC CAG TG | TaqMan probe |
| 5’- *mga* | GCGTTTGATAGCATCAAACAAGA | TaqMan primer |
| 3’- *mga* | CATCAAGGAGATGAACCCAGTTG | TaqMan primer |
| *mga* | TCACTTTTCGACAGCCCGTTGGTGA | TaqMan probe |
| 5’- *emm1* | AGA CAGTTACCATCAACAGGTGAAAC | TaqMan primer |
| 3’- *emm1* | TACTCCAGCTGTTGCCATAACAG | TaqMan primer |
| *emm1* | TAACCCATTCTTCACAGCGGCAGCC | TaqMan probe |
| 5’- *emm3* | TTCACGTGATGCGTCCAAGTC | TaqMan primer |
| 3’- *emm3* | CGTAAGGGTCTTCGCCGTGA | TaqMan primer |
| *emm3* | ACGCAAGCCGTCAAGGTCTTCGCCGT | TaqMan probe |
| 5’- *scpA* | CGAAAGAACCTTACCGCCTAGA | TaqMan primer |
| 3’- *scpA* | TTTACAATTTCGACACGCATCAA | TaqMan primer |
| *scpA* | GCAATTGAGCCTCAGGCATCGCA | TaqMan probe |
| 5’-*tufA* | CAACTCGTCACTATGCGCACAT | TaqMan primer |
| 3’-*tufA* | GAGCGGCACCAGTGATCAT | TaqMan primer |
| *tufA* | CTCCAGGACACGCGGACTACGTTAAAAA | TaqMan probe |
