# Supplementary material for: Phosphatase activity of the control of virulence sensor kinase CovS is critical for the pathogenesis of group A streptococcus
Source: PLoS Pathog. 2018 Oct 31;14(10):e1007354. doi: 10.1371/journal.ppat.1007354 (PMC6231683; doi:10.1371/journal.ppat.1007354)
Supplement: S2 Table — (DOCX) [file ppat.1007354.s002.docx]

**Table S2: Log_2_ fold changes of selected genes differentially regulated in the indicated strains compared to respective wild type strain**

| **Gene** | **M3-CovR-D53A** | **M3-CovS-E281A** | **M3-CovS-T284A** | **M1- CovR-D53A** | **M1- CovS-E281A** | **M1- CovS-T284A** |
| --- | --- | --- | --- | --- | --- | --- |
| *M5005_Spy1170* |  |  |  | -1.75 | -0.27 | -2.55 |
| *spd3* | - | - | - | -1.60 | -0.22 | -2.36 |
| *scpA* | 0.48 | 0.43 | -3.21 | 1.10 | 1.04 | -2.26 |
| *fba* | - | - | - | 0.99 | 0.94 | -2.14 |
| *nga* | 0.07 | 0.11 | -3.79 | 2.30 | 2.32 | -2.17 |
| *ifs* | 0.08 | 0.12 | -3.67 | 2.12 | 2.10 | -2.08 |
| *slo* | 0.10 | 0.14 | -3.64 | 2.14 | 2.13 | -2.07 |
| *ska* | 0.67 | 0.26 | -3.26 | 1.63 | 0.61 | -1.88 |
| *grm* | 0.32 | 0.25 | -2.36 | 1.05 | 0.88 | -1.79 |
| *sdaD2* | - | - | - | 1.23 | 1.34 | -1.56 |
| *sic* | - | - | - | 1.25 | 1.24 | -1.34 |
| *sclA* | 4.40 | 2.37 | -3.13 | 5.39 | 5.27 | -1.28 |
| *dppA* | 0.08 | 0.12 | -1.00 | 0.44 | 0.62 | -1.28 |
| *spyM3_0132** | 1.60 | 1.12 | -1.71 | 2.32 | 2.55 | -1.10 |
| *sagB* | -0.17 | -0.52 | -0.70 | 0.31 | -0.58 | -1.05 |
| *esterase* | 0.65 | 0.71 | -2.91 | -3.17 | 2.90 | -1.02 |
| *mga* | 0.10 | 0.13 | -1.12 | 0.07 | 0.10 | -1.01 |
| *spyA* | 0.39 | 0.38 | -2.31 | 2.16 | 2.17 | -0.97 |
| *bspA* | 0.04 | -0.11 | -1.19 | 0.22 | 0.06 | -0.81 |
| *hasA* | 0.97 | 0.79 | -4.80 | 4.62 | 4.16 | -0.31 |
| *prtS* | 2.02 | 1.79 | -4.48 | 4.74 | 4.57 | -0.25 |
| *mac-1* | 0.83 | 0.78 | -4.15 | 5.24 | 3.79 | -0.50 |
| *mf4* | 0.40 | 0.38 | -2.72 | - | - | - |
| *sla* | -0.27 | -0.19 | -2.57 | - | - | - |
| *spy_M3_0105*** | 1.91 | 1.61 | -2.52 | 4.35 | 3.82 | -0.47 |
| *speA* | 2.49 | 2.02 | -2.25 | - | - | - |
| *sdn* | -2.46 | -1.88 | -1.64 | - | - | - |
| *rofA* | 0.42 | 0.33 | -1.53 | 0.12 | 0.08 | 0.03 |
| *speK* | 0.04 | -0.01 | -1.43 | - | - | - |
| *emm* | 0.08 | 0.09 | -0.57 | 0.23 | 0.18 | -0.76 |

* M5005_*Spy0142; *** M5005_*Spy0115*
