# Supplementary figures and images for: Phosphatase activity of the control of virulence sensor kinase CovS is critical for the pathogenesis of group A streptococcus
Source: PLoS Pathog. 2018 Oct 31;14(10):e1007354. doi: 10.1371/journal.ppat.1007354 (PMC6231683; doi:10.1371/journal.ppat.1007354)

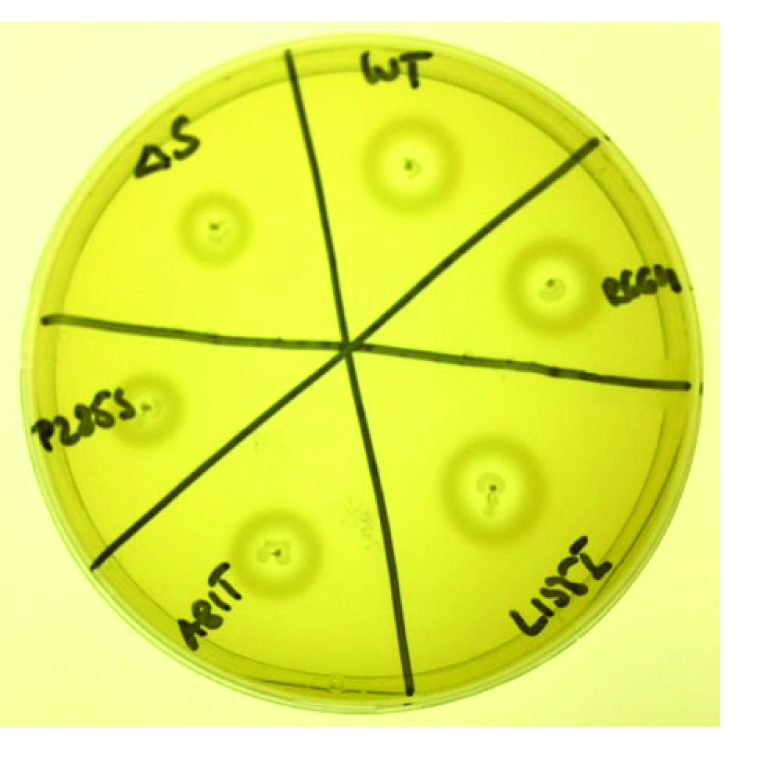

Supplement: S1 Fig — SpeB protease activity was assessed by the size of a clear zone around the bacterial growth on casein milk agar plates. Strains shown clockwise are WT = MGAS10870 (M3-WT), R66H = M3-CovR-R66H, L155I = M3-CovR-L155I, A81T = M3-CovR-A81T, P285S = M3-CovS-P285S, and ΔS = M3ΔcovS. (TIF) [file ppat.1007354.s003.tif]

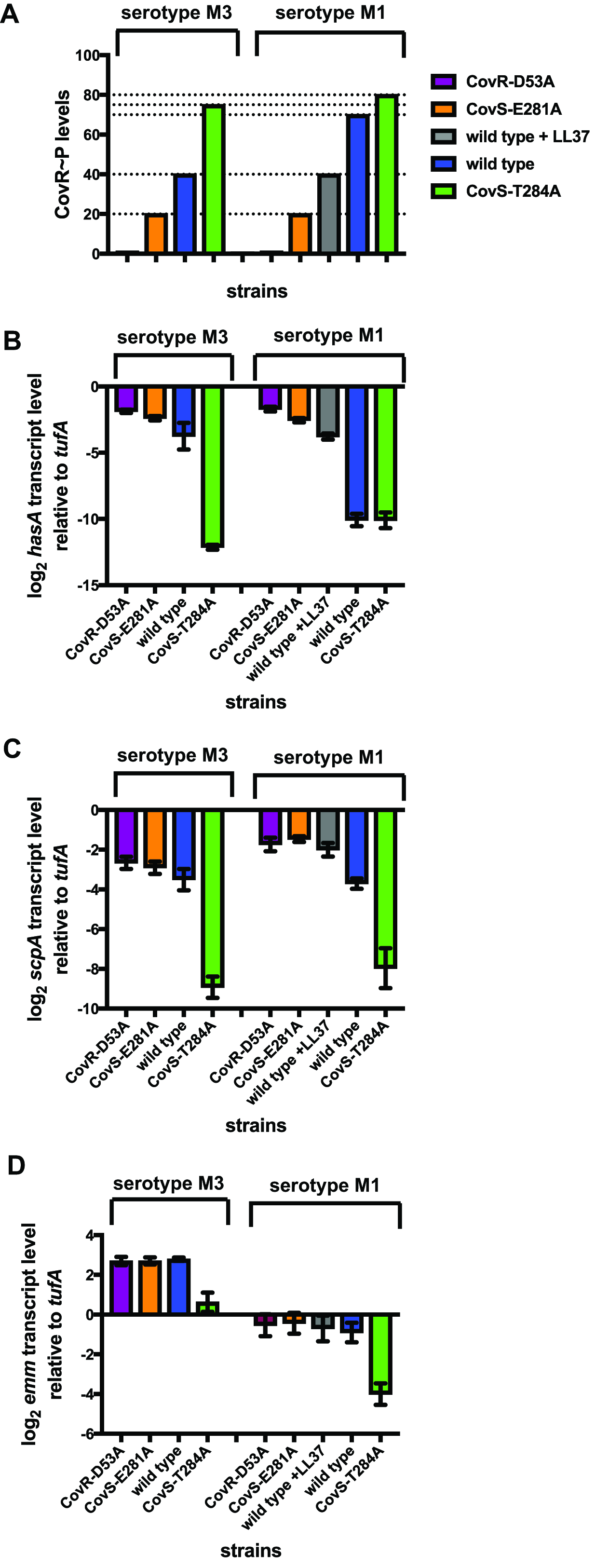

Supplement: S4 Fig — (A) Schematic depiction of CovR~P levels in the indicated GAS strains. Values were derived from previous Phostag-Western blot analyses of CovR~P status. (B-D) Transcript levels (means ± standard deviations; n = 4) of indicated genes that are representative for distinct gene classes in the isoallelic GAS strains relative to those of the wild type, as measured by TaqMan qRT-PCR. Strains were grown in THY to late-exponential phase. (TIF) [file ppat.1007354.s006.tif]
